# Which factors determine clinicians’ policy and attitudes towards medication and parent training for children with Attention-Deficit/Hyperactivity Disorder?

**DOI:** 10.1007/s00787-021-01735-4

**Published:** 2021-02-14

**Authors:** Tycho J. Dekkers, Annabeth P. Groenman, Lisa Wessels, Hanna Kovshoff, Pieter J. Hoekstra, Barbara J. van den Hoofdakker

**Affiliations:** 1 Department of Child and Adolescent Psychiatry, University of Groningen, University Medical Center Groningen, Groningen, the Netherlands; 2 Department of Psychology, University of Amsterdam, Amsterdam, the Netherlands; 3 Academic Center for Child and Adolescent Psychiatry and Specialized Youth Care, Amsterdam, the Netherlands; 4 Amsterdam UMC, Department of Child and Adolescent Psychiatry, Amsterdam, the Netherlands; 5 School of Psychology, University of Southampton, Southampton, UK

**Keywords:** Attention-deficit/hyperactivity disorder (ADHD), Children, Guidelines, Medication, Parent training, Psychosocial interventions

## Abstract

Behavioral parent and teacher training and stimulant medication are recommended interventions for children with attention-deficit/hyperactivity disorder (ADHD). However, not all children with ADHD receive this evidence-based care, and the aim of the current study was to find out why. More specifically, we investigated clinicians’ policy, guideline use, and attitudes towards medication and parent training when treating children with ADHD, as well as several factors that could affect this. A total of 219 Dutch clinicians (mainly psychologists, psychiatrists and educationalists) completed a survey. Clinicians were likely to recommend medication more often than parent training, and clinicians’ policy to recommend medication and parent training was positively associated with their attitudes towards these interventions. Less experienced clinicians and those with a non-medical background reported lower rates of guideline use, whereas clinicians with a medical background reported less positive attitudes towards parent training. Furthermore, a substantial portion of the clinicians based their decision to recommend parent training on their clinical judgement (e.g., prior estimations of efficacy, perceived low abilities/motivation of parents), and many clinicians reported barriers for referral to parent training, such as waiting lists or a lack of skilled staff. To achieve better implementation of evidence-based care for children with ADHD, guidelines should be communicated better towards clinicians. Researchers and policy-makers should further focus on barriers that prevent implementation of parent training, which are suggested by the discrepancy between clinicians’ overall positive attitude towards parent training and the relatively low extent to which clinicians actually advise parent training.

## Introduction

Attention-deficit/hyperactivity disorder (ADHD) is a highly prevalent neurodevelopmental disorder, characterized by excessive and impairing levels of inattention and/or hyperactivity-impulsivity [1, 2]. Relative to their unaffected peers, children with ADHD experience more learning [3] and social problems [4], and their quality of life is generally lower [5]. In the longer term, ADHD is associated with a range of adverse outcomes including substance abuse and antisocial behavior [6–8], and societal costs of ADHD are high [9, 10]. The high levels of impairment associated with untreated ADHD as well as the lower quality of life and adverse outcomes of affected children emphasize the importance of a wide implementation of effective interventions. Currently, not all children with ADHD receive effective treatment [11, 12], and the aim of the current study was to understand clinician-related factors that may represent barriers to evidence-based treatment for children with ADHD.

Many interventions exist for childhood ADHD, although most of these lack a solid evidence base. Effectiveness of stimulant medication, behavioral parent and teacher training, and the combination of medication and behavioral parent/teacher training is established [13, 14], whereas evidence for other interventions, such as neurofeedback, dietary interventions, cognitive training, and mindfulness, ranges from preliminary to non-existent [15–18]. Clinical guidelines summarize the scientific literature on the effectiveness of interventions, thereby disseminating and implementing scientific knowledge and translating this to recommendations for clinicians. Clinicians’ knowledge of, and adherence to guidelines is necessary to increase the use of effective interventions, thereby improving the quality of care for children with ADHD and their parents/caretakers, and reducing societal costs of ADHD [19].

Several guidelines for ADHD exist, which show considerable agreement. For children ages 6–12, all recognized guidelines recommend psychosocial interventions (i.e., behavioral parent or teacher training) and stimulant medication, although guidelines differ in their recommendation for pharmacological treatment as first-line treatment option as opposed to reserving this for more complex cases (e.g., high severity of problems, comorbidity) or only recommending medication after psychosocial interventions have not been effective [19–23].

In clinical practice, however, guidelines are not always followed. Although some clinicians primarily use rule-based approaches in making treatment-related decisions and are therefore mainly guideline-focused, others regard guidelines as vague, are less systematic, often base their decisions on idiosyncratic beliefs and experience, or are susceptible to external pressure by parents or teachers [24–26]. For example, a study on guideline use regarding clinical decisions about preschoolers with ADHD found that stimulant medication was part of the initial treatment plan for more than 60% of the preschoolers, despite guidelines stating that parent training is recommended as the first-line treatment option in this age group [27]. Given observations that one-third of children with ADHD who receive a low-intensity behavioral parent and teacher training as initial care do not require any further treatment [28], such deviations from guidelines can lead to unnecessary treatment. Another way in which non-adherence to guidelines can be problematic is when clinicians recommend interventions without a solid evidence base (e.g., mindfulness, neurofeedback, cognitive training), thereby potentially preventing children with ADHD and their families from receiving evidence-based care.

In the current study, we aimed to determine why many children with ADHD do not receive interventions as delineated in guidelines. We investigated associations between clinicians’ self-reported guideline use, their current policy, and their attitudes towards medication and parent training. Earlier research, for example, demonstrated that clinicians with a positive attitude towards psychosocial interventions demonstrated higher guideline adherence [24]. Therefore, these clinicians may discuss parent training more often in their current practice.

Clinicians’ policy to advise parent training and/or medication for children with ADHD may also be influenced by other characteristics. For example, experience of clinicians may be relevant for guideline use: less experienced clinicians appeared to adhere more to guidelines, whereas more experienced clinicians relied more on clinical judgement ([26] but see [27]). Also, clinicians with different professions may differ in guideline use, as well as in their attitudes towards medication and parent training. Potentially, clinicians with a medical background have a more positive attitude towards medication, which is tentatively suggested by findings that only 53% of the pediatricians routinely recommend behavioral interventions in case of childhood ADHD [29], and psychiatrists showing a relatively high preference for medication use in the long term [24]. On the other hand, clinicians with a non-medical background may also deviate from guidelines when, for example, their attitudes towards non-pharmacological treatment are positive.

Finally, we investigated possible barriers to clinicians referring parents of children with ADHD for parent training. First, a strong reliance on clinical judgement may prevent clinicians from advising parent training to certain parents of children with ADHD. Although relying on clinical judgement can be beneficial in individual cases, it is vulnerable to biases and cognitive distortions [26, 30, 31]. Second, a lack of knowledge about parent training could prevent clinicians from discussing parent training with parents, as limited knowledge about the content of interventions is an obstacle towards adhering to the guidelines that recommend these interventions [32, 33]. Third, practical barriers (e.g., a lack of trained staff, staff shortages, long waiting lists) may be associated with less frequent recommendations of parent training [25, 34].

## Methods

### Participants

Clinicians were recruited from 34 different Dutch institutions for child and adolescent mental health care. Inclusion criteria for these institutions were: (a) to perform diagnostic assessments of ADHD, (b) to provide parent training to parents of children with ADHD, (c) to prescribe medication for ADHD, and (d) to have at least 50 annual referrals of children for an ADHD assessment. Additionally, clinicians had to have annual involvement with at least 5 children with ADHD between 6 and 12 years old.

### Measures

Clinicians completed an online survey, which was constructed for the current study and was partially based on input from a focus group among clinicians. The survey consisted of general background questions (demographics, experience with ADHD, profession) followed by 24 items measuring constructs relevant to the current research questions, explained in detail below.

#### Guideline knowledge/use

Clinicians indicated their knowledge about the content of six ADHD guidelines (on a 5-point Likert scale: not at all, somewhat, reasonable, good, complete), and the extent to which they used these guidelines (on a 5-point Likert scale: never, rarely, sometimes, often, always). We selected three national Dutch guidelines: The Dutch multidisciplinary guideline ADHD in children and adolescents [35], the Dutch guideline for general practitioners (i.e., “NHG guideline”) [36], and the Dutch ADHD guideline for youth care and protection [37]. Furthermore, we selected three international guidelines that were often used in the Netherlands at the moment of the survey: guidelines of the American Academy of Child and Adolescent Psychiatry [20], the American Academy of Pediatrics [38], and the National Institute for Health and Care Excellence [39]. Clinicians reported on their knowledge and use of the six guidelines, and there was an “other” category in case clinicians used other guidelines. Although differences between these guidelines exist, the overlap is substantial and therefore the highest score on any of the six guidelines was used as outcome measure for guideline knowledge and guideline use, respectively. This yielded two outcomes for each clinician: one for guideline knowledge and one for guideline use. As these variables were highly correlated (*r*_*s*_ = .690, *p* < .001), we decided only to conduct further analyses on guideline use as outcome variable.

#### Attitude towards parent training or medication

To estimate clinicians’ attitudes towards parent training and medication, clinicians rated two statements (*“I have a strong preference for parent training as treatment for ADHD”* and *“I have a strong preference for medication as treatment for ADHD”*) on a 5-point Likert scale, ranging from totally disagree to totally agree.

#### Policy: discussing parent training/medication

Clinicians indicated in what percentage of the children with ADHD between 6 and 12 years they discuss parent training and medication as treatment options on two items (i.e., one for parent training and one for medication).

#### Clinical judgement

Clinicians rated the degree to which they use their clinical judgement when advising parent training to parents of children with ADHD ages 6–12 on four items, using a 5-point Likert scale (totally disagree to totally agree): *“I do not advise parent training in case of complex family situations (e.g., divorced parents, multi-problem families, parental psychopathology, severe psychosocial problems)”*, *“I try to make my own estimation whether parent training will work with that particular parents and base my advice upon that”*, *“Parent training is less suitable for parents that do not have enough abilities to apply the learned techniques, and therefore I don’t advise it in these cases”,* and *“I only advise parent training to parents if I think they are sufficiently motivated”*. The sum score of these four items was interpreted as measure of clinical judgement.

#### Lack of knowledge about parent training

Clinicians indicated on a 5-point Likert scale (totally disagree to totally agree) whether a lack of knowledge about parent training causes them not to advise parent training regularly as treatment for children with ADHD, ages 6-12: *“I don’t often advise parent training as treatment option for children with ADHD because my knowledge about parent training is limited”*.

#### Practical barriers

Clinicians rated the degree to which they experience practical barriers prevent them from recommending parent training to parents of children with ADHD ages 6–12 on three items using a 5-point Likert scale (totally disagree to totally agree): *“In my institution, there is not enough skilled staff to offer parent training as treatment for ADHD”*, *“If parent training is practically not feasible for parents (e.g., no one to take care of children, no car), I will not advise this option”*, and *“If waiting lists for parent training are long, I advise a different form of treatment because of that”*. The sum score of these three items was interpreted as measure of practical barriers for advising parent training.

### Procedure

Dutch clinicians working in participating mental health care institutions received an email with a link to the online survey. Completing the survey implied active consent. Surveys were administered in 2017 or 2018.

### Data analysis

A three-tiered data-analytic approach was adopted. In Tier I, we used hierarchical bootstrapped regression analyses (with bias-corrected 95% confidence intervals, 1000 bootstrapping samples) to test which factors were associated with clinicians’ policy regarding parent training and medication. The latter were used as outcome variables in two separate regression analyses. For both analyses, guideline use was tested as predictor in the first block. In the second block, clinicians’ attitude towards either parent training or medication was added to the regression model.

In Tier II, we examined which characteristics of clinicians (i.e., sex, experience in working with ADHD, profession) were related to their guideline use, their attitudes and their policy. Potential sex differences were tested with chi-square analyses. Differences in guideline use, attitudes and policy between the four groups based on experience in working with children with ADHD were assessed with Kruskal–Wallis tests with non-parametric Bonferroni-adjusted follow-up pairwise comparisons. Differences in guideline use, attitudes and policy between professionals with medical and non-medical backgrounds were compared using Mann–Whitney tests.

In Tier III, we assessed which factors related to parent training (i.e., clinical judgement, lack of knowledge about parent training, practical barriers) were associated with clinicians’ guideline use, and with their attitudes and policy regarding parent training. A bootstrapped multiple regression analysis containing all factors (i.e., clinical judgement, lack of knowledge about parent training, practical barriers) was used for each outcome measure (i.e., guideline use, attitude towards parent training and policy regarding parent training).

For all analyses, bootstrapped or non-parametric tests were selected because the assumption of normality was violated for all outcome measures, as indicated by significant Kolmogorov–Smirnov tests. All analyses were performed is SPSS version 25, using an alpha of .05.

## Results

### Descriptive information

A total of 328 clinicians started the survey. From this sample, 109 were excluded as they indicated that they were involved with fewer than 5 children with ADHD annually, and therefore the final sample consisted of 219 clinicians. As delineated in Table 1, the sample was diverse in terms of age, experience with ADHD and profession. A post hoc power analysis using G*Power [40] further indicated that the current sample was adequately powered (1 – *β* = .99) to detect effects of medium magnitude (given a linear multiple regression analysis with three predictors, and *α* = .05).

**Table 1:** Demographic and profession-related characteristics of participating clinicians.

		*N* = 219 (%)
Sex	Male	14.6
	Female	85.4
Age	20–29 years	25.1
	30–39 years	38.4
	40–49 years	18.3
	50–59 years	14.6
	≥ 60 years	3.7
Experience with ADHD	< 2 years	19.2
	2-5 years	20.2
	5-10 years	28.8
	> 10 years	31.8
Profession	Psychiatrist	5.0
	Psychologist	36.5
	Educationalist	22.4
	Nurse	5.9
	Physician (non-psychiatrist)	2.7
	Social worker	3.7
	Expressive therapist	2.7
	Other/multiple	21.0
Medical Profession	Yes	16.9
	No	83.1

### Tier I: What factors determine clinicians’ policy? Associations with clinicians’ guideline use and attitudes

Response distributions on clinicians’ guideline use and their attitudes and policy regarding ADHD treatment are presented in Table 2. More than 40% of clinicians indicated that they used ADHD guidelines ‘only sometimes’ or less often than this. Generally, clinicians had a neutral to positive attitude towards parent training, whereas their attitude towards medication was more negative. In their daily practice, almost one-third of the clinicians recommended parent training to only a minority of the parents of children with ADHD they treated, whereas the majority of clinicians considered medication in most cases.

**Table 2:** Clinicians’ guideline use and their attitudes and policies regarding ADHD treatment.

Guideline use	Never	15.1%
	Rarely	6.8%
	Sometimes	18.3%
	Often	42.9%
	Always	16.9%
Attitude: preference for parent training	Totally disagree	0.5%
	Disagree	6.4%
	Neutral	26.9%
	Agree	49.3%
	Totally agree	16.9%
Attitude: preference for medication	Totally disagree	8.7%
	Disagree	46.6%
	Neutral	36.5%
	Agree	7.8%
	Totally agree	0.5%
Policy: discuss parent training as potential treatment	0-20% of children	12.8%
	20-40% of children	16.0%
	40-60% of children	17.8%
	60-80% of children	21.9%
	80-100% of children	31.5%
Policy: discuss medication as potential treatment	0-20% of children	8.7%
	20-40% of children	3.2%
	40-60% of children	11.0%
	60-80% of children	21.9%
	80-100% of children	55.3%

#### Policy: parent training

Guideline use was associated with clinicians’ tendency to discuss parent training as a treatment option, *β* = .261, bootstrapped 95% CI [.109, .410], *p* = .001. In the second block of the regression analysis, with clinicians’ attitude towards parent training added to the model, both guideline use and clinicians’ attitude towards parent training were associated with clinicians’ policy of discussing parent training as treatment option, *β* = .219, bootstrapped 95% CI [.065, .369], *p* = .004 and *β* = .254, bootstrapped 95% CI [.196, .575], *p* = .001, respectively.

#### Policy: medication

Guideline use was associated with clinicians’ tendency to discuss medication as a treatment option, *β* = .188, bootstrapped 95% CI [.061, .345], *p* = .006. In the second block of the regression analysis, with clinicians’ attitude towards medication added to the model, both guideline use and clinicians’ attitude towards medication were associated with clinicians’ policy to discuss medication as treatment option, *β* = .177, bootstrapped 95% CI [.043, .336], *p* = .010 and *β* = .298, bootstrapped 95% CI [.307, .768], *p* = .001, respectively.

### Tier II: Which clinician characteristics are related to their guideline use, attitudes and policy?

#### Sex

Male and female clinicians did not differ in their stated guideline use (*χ*^*2*^(4) = .62, *p* = .96), in their attitudes towards parent training and medication (*χ*^*2*^(4) = 5.82, *p* = .21 and *χ*^*2*^(4) = 8.52, *p* = .07, respectively), or in their policy to discuss parent training and medication as potential treatment options (*χ*^*2*^(4) = 4.66, *p* = .32 and *χ*^*2*^(4) = 5.64, *p* = .23, respectively).

#### Experience with ADHD

Experience in treating children with ADHD was positively associated with guideline use, *H*(3) = 9.470, *p* = .024. Bonferroni-adjusted non-parametric follow-up analyses indicated that clinicians with more than 10 years of experience working with children with ADHD indicated that they used guidelines more often than clinicians with less than 2 years of experience, *p* = .020, *r* = .293^1^. Other pairwise comparisons between the experience-based categories in relation to guideline use were not significant. Other effect sizes, although not statistically significant, confirmed the pattern that clinicians with less than 2 years of experience with ADHD used guidelines less frequently than more experienced clinicians (*r* = .19 and *r* = .25, as compared with clinicians with 2–5 and 5–10 years of experience with ADHD, respectively).

Experience working with children with ADHD was not associated with attitudes towards parent training, *H*(3) = 2.770, *p* = .428, but was related to attitudes towards medication, *H*(3) = 13.118, *p* = .004. Bonferroni-adjusted non-parametric follow-up analyses indicated that clinicians with more than 10 years of experience working with children with ADHD had more positive attitudes towards medication than clinicians with less than 2 years and clinicians with 5–10 years of experience, *p* = .009, *r* = .315 and *p* = .032, *r* = .275, respectively. Other pairwise comparisons between the experience-based categories in relation to attitudes towards medication were non-significant.

Furthermore, ADHD-related experience was not associated with clinicians’ reports of their tendency to discuss parent training as treatment option, *H*(3) = 3.191, *p* = .363, or with their policy to discuss medication as treatment option, *H*(3) = 6.987, *p* = .072.

#### Profession

Clinicians with a medical profession indicated that they used guidelines more often than clinicians with a non-medical profession, *U* = 4576.0, *z* = 3.614, *p* < .001, *r* = .244. Furthermore, clinicians with a medical background did not differ from clinicians without a medical background in their attitude towards medication, *U* = 3965.5, *z* = 1.849, *p* = .065, *r* = .125. Clinicians without a medical background, however, had a more positive attitude towards parent training than clinicians with a medical profession, *U* = 2205.5, *z* = -3.574, *p* < .001, *r* = − .242.

With regard to clinicians’ policies, medical professionals did not discuss medication as a potential treatment option more often than clinicians without a medical background, *U* = 3983.0, *z* = 1.802, *p* = .072, *r* = .122, and similarly, both groups were equally likely to discuss parent training as potential treatment option, *U* = 3371.5, *z* = .014, *p* = .989, *r* = .001.

### Tier III: Do clinical judgement, lack of knowledge about parent training, and practical barriers influence clinicians’ guideline use and their preference and policies regarding parent training?

Response distributions on the items pertaining to clinical judgement, lack of knowledge about parent training, and practical barriers are presented in Table 3. A substantial portion of the clinicians based their advice regarding parent training upon their clinical judgement: more than one-third of the clinicians based their advice upon their own estimations of the efficacy of parent training, and more than one-fifth of the clinicians indicated that parent training was less suitable for parents with lower abilities. Also, parents’ perceived lack of motivation was endorsed as a reason not to advise parent training for more than one-fifth of clinicians. A small proportion of clinicians (<10%) indicated that the complexity of the family situation was a reason not to advise parent training.

Furthermore, only a very small number of clinicians indicated that they did not advise parent training because their knowledge about parent training was insufficient. Finally, almost one-fifth of clinicians indicated that their institution/clinic did not have enough skilled staff for parent training, and almost one-third of the clinicians indicated that long waiting lists for parent training led them to advise different forms of treatment. Practical problems experienced by parents only prevented a very small number of clinicians from advising parent training.

**Table 3:** Response distribution on use of clinical judgement, lack of knowledge, and practical barriers – all in relation to parent training.

		*N* = 219
**Clinical judgement**		
*“I do not advise parent training in case of complex family situations”*	Totally disagree Disagree Neutral Agree Totally agree	15.5% 50.2% 25.1% 8.7% 0.5%
*“I try to make my own estimation whether parent training will work with that particular parents and base my advice upon that”*	Totally disagree Disagree Neutral Agree Totally agree	6.4% 31.1% 26.5% 32.9% 3.2%
*“Parent training is less suitable for parents that do not have enough abilities to apply the learned techniques, and therefore I don’t advise it in these cases”*	Totally disagree Disagree Neutral Agree Totally agree	5.9% 42.5% 29.2% 21.5% 0.9%
*“I only advise parent training to parents if I think they are sufficiently motivated”*	Totally disagree Disagree Neutral Agree Totally agree	5.5% 47.0% 25.1% 20.5% 1.8%
**Lack of knowledge about parent training**		
*“I don’t often advise parent training as treatment option for children with ADHD because my knowledge about parent training is limited”*	Totally disagree Disagree Neutral Agree Totally agree	62.1% 31.5% 4.1% 2.3% 0%
**Practical barriers**		
*“In my institution, there is not enough skilled staff to offer parent training as treatment for ADHD”*	Totally disagree Disagree Neutral Agree Totally agree	29.7% 37.0% 13.7% 13.7% 5.9%
*“If parent training is practically not feasible for parents (e.g., no one to take care of children, no car), I will not advise this option”*	Totally disagree Disagree Neutral Agree Totally agree	26.9% 59.8% 12.3% 0.9% 0%
*“If waiting lists for parent training are long, I advise a different form of treatment because of that”*	Totally disagree Disagree Neutral Agree Totally agree	6.4% 25.1% 37.4% 29.2% 1.8%

#### Guideline use

Clinicians’ limited knowledge of parent training was associated with poor use of clinical guidelines, *β* = − .331, bootstrapped 95% CI [− .845, − .339], *p* = .001. In the same regression model, clinical judgement and practical barriers were not associated with guideline use, *β* = − .076, bootstrapped 95% CI [-.114, .033], *p* = .280 and *β* = .094, bootstrapped 95% CI [-.031, .176], *p* = .208, respectively.

#### Attitude towards parent training

Clinicians’ report of having insufficient knowledge about parent training was associated with negative attitudes towards parent training, *β* = -.304, bootstrapped 95% CI [− .504, − .205], *p* = .001. Again, in the same regression model, clinical judgement and practical barriers were not associated with preference for parent training, *β* = − .045, bootstrapped 95% CI [− .063, .031], *p* = .505 and *β* = − .071, bootstrapped 95% CI [− .095, .025], *p* = .291, respectively.

#### Policy: discussing parent training as treatment option

Clinicians’ limited knowledge of parent training was associated with discussing parent training as a treatment option less frequently *β* = − .442, bootstrapped 95% CI [− 1.058, − .522], *p* = .001. Again, in the same regression model, clinical judgement and practical barriers were not associated with policies regarding parent training, *β* = − .082, bootstrapped 95% CI [− .110, .022], *p* = .195 and *β* = .081, bootstrapped 95% CI [− .035, .140], *p* = .231, respectively.

## Discussion

The aim of this study was to examine barriers to guideline-informed clinical treatment decisions for children with ADHD between 6 and 12 years old. In particular, we were interested to understand factors that were associated with clinical recommendations of parent training and/or medication [11, 12], which were—and still are—advised as first-line interventions for children with ADHD^2^ [19, 20, 23]. Wider dissemination and implementation of evidence-based interventions for children with ADHD may be achieved by gaining more knowledge about factors related to clinicians’ use of guidelines, attitudes towards different types of interventions, and clinical practices. This benefits the well-being of children with ADHD and their relatives, and can reduce societal costs [9, 10].

The first main finding of the current study was that clinicians reported that their tendency to recommend parent training and medication as treatment options were associated with their attitudes towards these interventions, even after controlling for self-reported guideline use. Clinicians with more positive attitudes towards parent training recommended the use of parent training more often, and similarly, clinicians with more positive attitudes towards medication were more likely to advise the use of medication more often as a first-line treatment option. These findings suggest that targeting clinicians’ attitudes towards specific interventions could lead to meaningful change in their policy.

The second main finding was that years of experience of working with children with ADHD as well as professional background were related to clinicians’ guideline use. These factors were also associated with attitudes towards medication and parent training and the tendency to discuss these interventions as treatment options. More specifically, clinicians with less than two years of experience working with children with ADHD were less likely to use clinical guidelines relative to their more experienced colleagues. This seems at odds with previous studies demonstrating that inexperienced clinicians rely *more* heavily on guidelines, whereas more experienced clinicians are more likely to rely on pattern recognition [26, 41]. However, the association between inexperience and lower guideline use in the current study was particularly driven by a very inexperienced (i.e., less than two years of experience) subgroup of clinicians. This low level of experience could imply that these clinicians may have had fewer opportunities to learn about the content of guidelines, or that there is a lack of emphasis on the importance of guideline use in the education of clinicians. An alternative, opposite explanation is also possible: the content of the training completed recently by inexperienced clinicians could overlap with the content of guidelines, causing a lower sense of urgency to utilize guidelines in these clinicians. Furthermore, clinicians with more than ten years of experience with ADHD had a more positive attitude towards medication than their less experienced colleagues. Clinicians with a medical profession indicated that they were more likely to use guidelines to inform their policy, but they also reported a more negative attitude towards parent training than clinicians with a non-medical background, which is in line with earlier research [29].

The third main finding was that lower knowledge about parent training negatively influenced clinicians’ guideline use, their attitude towards parent training, and their policy to discuss parent training. However, although lower levels of knowledge about the content of interventions have been related to lower guideline use in previous research [32, 33], the current findings should be viewed with more nuance. Inspections of our findings suggest that the effect of lack of knowledge was mainly driven by clinicians either disagreeing or totally disagreeing to the statement “I don’t often advise parent training as treatment option for children with ADHD because my knowledge about parent training is limited”.

Although unrelated to guideline use, attitudes or policies, it was notable that a substantial proportion of clinicians in the current study used clinical judgement when deciding whether or not to advise parent training (i.e., making prior estimations of efficacy, taking abilities and motivation of parents into account). Relying on clinical judgement can be beneficial in individual cases, but is also vulnerable to bias and cognitive distortion [26, 30, 31]. Future studies are needed to elucidate whether the factors that clinicians intuitively take into account are related to lower effectiveness of parent training (e.g., is parent training less effective when parents are unmotivated or have lower abilities?), but the current evidence does not specify such conditions: The effectiveness of parent training does not seem lower for parents with mental health problems, and parent training is not contraindicated in case of complex family problems [42]. Parent training is therefore recommended as preferred treatment for *all* children with ADHD.

### Strengths and limitations

A strength of the current investigation is that we included a large, diverse, and representative sample of clinicians working in child mental health care. A notable limitation is that all established relations were correlational, which may serve to fuel future studies that can elucidate causal relations. Longitudinal studies could investigate whether increasing awareness for guideline use in general, and for parent training as treatment option for ADHD in particular, for example, by changing education curricula, ultimately improves clinicians’ practices when working with children with ADHD.

A second limitation is that the extent to which the results of this study generalize to other countries depends on mental healthcare systems, which may vary between countries. Furthermore, no general practitioners or pediatricians participated in our study, which may limit generalization as well.

### Implications

A first implication of this study is that guideline use among clinicians should be increased as this was found to be low (i.e., more than 40% of clinicians reported using ADHD guidelines ‘sometimes’, or less). Many clinicians deviated from these guidelines in their daily practice, which was most obvious with regard to parent training: about one-third of the clinicians only advised the use of parent training for a minority of the children with ADHD they were treating, whereas guidelines uniformly recommend parent training as preferred first-choice treatment for all children with ADHD [20, 38, 39]. Therefore, guideline use among clinicians should be encouraged, for example, by offering guideline training in education programs for both medical and non-medical professions. Efforts in this direction could be particularly focused on clinicians with low levels of experience in working with ADHD, as well as clinicians with a non-medical background, as these subgroups indicated lower guideline use. Although difficulties in the implementation of guidelines are widely described [33, 43, 44], it was demonstrated that an assessment of clinicians’ guideline adherence combined with a multi-day training had positive effects on their subsequent practices [45]. Another potential way to increase guideline use is to improve the ease of applicability of these guidelines, for example, by developing an app with guideline-based decision trees or guideline checks embedded in electronic patient records, as clinicians often experience guidelines as vague and non-specific [25].

A second implication of this investigation is that the effectiveness of parent training as intervention for ADHD should receive more attention in educational programs of child mental health care. We found that relative to medication, clinicians endorsed the use of parent training substantially less often. This was surprising as (i) the same clinicians reported more positive attitudes towards parent training than towards medication and (ii) a large body of research indicates that parents often prefer psychosocial interventions as a first-line treatment in favor of medication [46, 47].

Third, broad, inexpensive, fast and flexible availability of parent training should be a core priority for policy-makers in child mental health care, as we found that factors beyond control of the individual clinician also prevented them from advising parent training. In the current study, almost 20% of the clinicians reported a shortage of skilled staff available for parent training in their clinic and almost one-third indicated that they were unlikely to recommend parent training because of long waiting lists. Previous studies demonstrated that parent training was often not recommended because it was unavailable [27]. Parent-related barriers are also likely, as participating in an 8–10 session parent training program could be problematic for different reasons (e.g., practically, financially, too time-consuming) [48–50].

Fourth, increasing knowledge about parent training should be a priority. While parent training is recommended by all major ADHD guidelines, its content is not always clearly specified, and largely differs across studies and intervention models [42]. Relative to medication, for which specific step-by-step protocols exist [14, 51], there is a lack of uniformity regarding the content of parent training, which is reflected by the existence of a plethora of different parent training protocols [52]. This could be confusing for clinicians and improvements in this respect are likely to improve the implementation of parent training in the care for children with ADHD [15]. Additionally, communication between parent training therapists and clinicians responsible for assessment of ADHD could potentially be improved. If parent training therapists and clinicians more closely collaborate, clinicians are likely to receive more information about parent training which could stimulate them to recommend parent training more often.

In sum, many factors seem to explain why children with ADHD do not always receive guideline-focused evidence-based care and potential opportunities for improvement in clinical practice may be achieved through education of clinicians, but also by system-related changes which should facilitate easier application of evidence-based interventions.

## Data Availability

Data cannot be made publicly available, as participants did not give consent for this.

## Footnotes

1 The effect size of the follow-up pairwise comparison of the Kruskal-Wallis was calculated by: $r=\frac{z}{\sqrt{n}}$ [53].

2 Note that the recent NICE guidelines (2018) only recommend parent training in case of comorbid symptoms of ODD/CD.
